# Cost–utility analysis of Social Stories™ for children with autism spectrum disorder in mainstream primary schools: results from a randomised controlled trial

**DOI:** 10.1192/bjo.2024.47

**Published:** 2024-06-03

**Authors:** Han-I. Wang, Kerry Bell, Jane Blackwell, Charlie Welch, Laura Mandefield, Judith Watson, Emma Standley, Dean McMillan, Simon Gilbody, Barry Wright, Catherine Hewitt, Steve Parrott

**Affiliations:** Department of Health Sciences, University of York, UK; and York Trial Unit, Department of Health Sciences, University of York, UK; Department of Health Sciences, University of York, UK; and Hull York Medical School, University of York, UK; Department of Health Sciences, University of York, UK; Hull York Medical School, University of York, UK; and Child Oriented Mental Health Intervention Centre (COMIC), Leeds and York Partnership NHS Foundation Trust, York, UK

**Keywords:** Autism spectrum disorders, Social Stories, child and adolescent, cost-effectiveness, quality-adjusted life-years

## Abstract

**Background:**

One in 57 children are diagnosed with autism in the UK, and the estimated cost for supporting these children in education is substantial. Social Stories™ is a promising and widely used intervention for supporting children with autism in schools and families. It is believed that Social Stories™ can provide meaningful social information to children that can improve social understanding and may reduce anxiety. However, no economic evaluation of Social Stories has been conducted.

**Aims:**

To assess the cost-effectiveness of Social Stories through Autism Spectrum Social Stories in Schools Trial 2, a multi-site, pragmatic, cluster-randomised controlled trial.

**Method:**

Children with autism who were aged 4–11 years were recruited and randomised (*N* = 249). Costs measured from the societal perspective and quality-adjusted life-years (QALYs) measured by the EQ-5D-Y-3L proxy were collected at baseline and at 6-month follow-up for primary analysis. The incremental cost-effectiveness ratio was calculated, and the uncertainty around incremental cost-effectiveness ratios was captured by non-parametric bootstrapping. Sensitivity analyses were performed to evaluate the robustness of the primary findings.

**Results:**

Social Stories is likely to result in a small cost savings (–£191 per child, 95% CI −767.7 to 337.7) and maintain similar QALY improvements compared with usual care. The probability of Social Stories being a preferred option is 75% if society is willing to pay £20 000 per QALY gained. The sensitivity analysis results aligned with the main study outcomes.

**Conclusions:**

Compared with usual care, Social Stories did not lead to an increase in costs and maintained similar QALY improvements for primary-aged children with autism.

Autism spectrum disorder is a lifelong neurodevelopmental condition that includes differences in the way children experience the world, such as social communication differences that can lead to stress in interactions.^1^ In the UK, one in 57 children are diagnosed with autism,^2^ and the estimated cost of supporting these children is around £3.1–3.4 billion (in 2011 value) per year, with special educational needs being the main cost driver (47%), followed by reported parental productivity loss as they care for their children (12%).^3^

Given this significant cost in education systems and limited funding available for specialist support in schools,^4^ it is important that clinically effective interventions that are also cost-effective can be delivered within schools on a day-to-day basis; that they are safe and child-centred; and that they are tailored to a neurodiverse population. Currently, only one relevant economic evaluation study was done;^5^ the study suggests that the LEGO®-based therapy delivered in a school setting is likely to lead to a small cost saving and small improvements in quality-adjusted life-years (QALYs) compared with usual care.

## Study aims

Carol Gray's Social Stories™ is a promising intervention that can potentially alleviate the social communication difficulties,^6,8^ as well as not being costly, intrusive, time-consuming or requiring extensive involvement of outside experts.^9^ Such positive outcomes and features make the use of Social Stories very popular in schools and in families with children with autism.^10^ Despite the growing interest, there is a lack of economic evaluation study for Social Stories. Therefore, this study aims to assess the cost-effectiveness of Social Stories alongside usual care for children with autism in primary schools, compared with usual care alone. This paper reports the economic evaluation results of Social Stories alongside the Autism Spectrum Social Stories in Schools Trial 2 (ASSSIST-2) trial, and follows the Consolidated Health Economic Evaluation Reporting Standards 2022.^11^

## Method

### Trial design and participants

This economic evaluation was incorporated into the ASSSIST-2 trial, a pragmatic, two-arm, cluster-randomised controlled trial conducted at the school level. The trial compared the cost-effectiveness of Social Stories plus usual care with usual care alone, for primary-aged children with autism. The protocol for the ASSSIST-2 trial has been published elsewhere.^12^ In brief, children diagnosed with autism spectrum disorder and aged between 4 and 11 years were recruited from mainstream primary schools in Yorkshire and Humber from November 2018 to May 2021. Parents/guardians and schools were approached to discuss eligibility and provide consent. The detailed inclusion and exclusion criteria can be found in Supplementary File 1, Appendix 1 available at https://doi.org/10.1192/bjo.2024.47. Participating school clusters were randomised in a 1:1 ratio to either the intervention or control condition, using blocked randomisation. Stratification was performed based on school type (special educational needs school or mainstream school) and the number of participating children within the school (five or fewer or more than five participating children). To mitigate against selection bias, all participating children were recruited, and had their baseline assessments completed before school randomisation. Children with autism who were assigned to the intervention arm received Social Stories alongside their usual care and education. Children with autism who were assigned to the control arm received usual care and education only, comprising the routine support typically offered to children with autism by educational and health services. Follow-up assessment was conducted for all children with autism up to 6 months post-randomisation. Refer to Supplementary File 1, Appendix 2 for a flowchart detailing the study.

### Intervention

Social Stories was developed by Carol Gray, a specialist teacher, in 1993.^13^ It comprises a collection of short stories that usually write the child with autism into a story that includes them in a social situation. They describe, in positive and friendly language, social information about a situation, which the child may be missing or need to know. This can help the child in that situation and can reduce anxiety.^9^ In the current trial, the intervention included training for interventionists and parents/guardians covering autism psychoeducation, design and implementation of Social Stories. Stories were then developed around specific goals that were agreed by teachers, interventionists and parents/guardians to address the child's need for social information. To deliver the intervention, interventionists read the Social Story with the child at least six times over 4 weeks, during school hours, with some level of flexibility to make changes in delivery^12^ depending on factors such as accessibility for the child and logistical issues at school.

### Health outcome measurement

The health outcomes were QALYs measured by the EQ-5D-Y-3L (proxy version).^14^ The EQ-5D-Y-3L (proxy version) is a generic preference-based tool assessing health-related quality of life with three severity levels, ranging from 1 (indicating the best health) to 3 (indicating the worst health) over five dimensions/domains (mobility, looking after themselves, doing usual activities, having any pain or discomfort, and feeling worried or sad). This measure allows a proxy person, such as a parent or guardian, to complete it on behalf of children.^14^ The EQ-5D-Y-3L has demonstrated reliability and validity as an instrument for assessing health-related quality of life in children^15^ and individuals with a broader category of neurodevelopmental differences, including attention-deficit hyperactivity disorder,^16^ speech/language disorder^17^ and functional disability,^18^ suggesting a reasonable basis for EQ-5D-Y-3L's applicability to children and young people with autism.

Responses at the individual level to the EQ-5D-Y-3L proxy were utilised to calculate utility scores, using the Dutch EQ-5D-Y-3L value set.^19^ A utility represents a child's health state ‘today’, and ranges from less than 0 (worse than death) to 0 (death) and 1 (full health). The utilities measured at different time points were aggregated through the area under the curve technique, to compute QALYs up to 6 months post-randomisation.^20^

### Cost measurement

#### Costs of the Social Stories intervention

Costs associated with the Social Stories intervention encompassed both training costs and the cost related to delivering the intervention. Training costs included preparation time, time spent on delivering training sessions, travel expenses and the material costs during the training. Costs related to delivering the Social Stories intervention included the time spent by professionals to plan and conduct sessions, and the material costs. A bottom-up costing approach was applied,^20^ and the staff time costs were estimated by the time spent and the costs per minute, based on salaries. The study team collected all relevant data through the tailored questionnaires.

#### Cost of service use

Data on service use were gathered through two tailored questionnaires^12^ developed by the research team, based on our previously successful school-based trial, the Investigating Social Competence and Isolation in Children with Autism taking part in LEGO-based Therapy Clubs in School Environments (I-SOCIALISE) trial.^5^ The questionnaire completed by parents/guardians collects information on healthcare services utilisation, including both hospital-based services and those outside a hospital setting, such as community health services, services provided by allied health professionals, mental health services and social services. It also covers school-based services, including those offered by educational psychologists and specialist teacher advisors, as well as parental private expenses and productivity loss. The questionnaire completed by teachers gathers information regarding intervention and support within the school settings, as well as the impact of children's behaviour on school resources. The resource use questionnaires for parents/guardians and teachers are available in Supplementary File 2.

To determine the total cost for each arm, we employed the bottom-up costing approach.^20^ The quantity of service use was multiplied by the respective unit costs. Unit costs for healthcare service use were sourced from the 2019/20 National Cost Collection^21^ and the Unit Costs of Health and Social Care Report 2019.^22^ Medication costs were derived from the Prescription Cost Analysis – England 2019,^23^ whereas teacher time costs were estimated with data from the Department for Education's 2019 Report.^24^ Privately paid services were estimated based on market prices, and productivity loss was assessed based on national average wage rates. All costs were expressed in British pounds for the UK financial year 2019–2020. No discount rate was used, as the study time horizon was less than a year (6 months). The list of unit costs is presented in Supplementary File 1, Appendix 3.

### Missing data

In the current study, the term ‘complete case’ refers to children with comprehensive utility and cost data at all time points, whereas the ‘base case’ refers to children with missing utility and/or cost data at follow-up, despite having complete baseline assessments. The multiple imputation method via chained equations^25^ was employed to impute the identified missing utility and cost data. This imputation process considered trial arm, age, gender, stratification factors (special educational needs status and number of participants), baseline parent-completed Social Responsiveness Scale, Second Edition (SRS-2) score, baseline utility and baseline costs from the societal perspective.

### Statistical and economic analysis

From the societal perspective, the primary analysis estimated the incremental cost-effectiveness ratio (ICER), taking into consideration healthcare service costs, education-related service costs, parental out-of-pocket costs (including childcare and private courses) and productivity loss for parents, representing time off work because of their child's autism.

To account for uncertainty around the ICER and imbalanced utility and costs at baseline, a multivariate multilevel model (MMLM) adjusted for clustering and controlled for baseline utility,^26^ and baseline costs from the societal perspective, age, gender and baseline parent-completed SRS-2 scores were conducted and bootstrapped 5000 times. The application of the MMLM method managed both cost and QALY distributions and addressed their correlation.^27^ Also, the non-parametric cluster-level bootstrap resampling method was used, considering the likely skewness in the distribution of regression residuals.^28^ To analyse the imputed data using bootstrapping, we followed the approach proposed by Leurent and colleagues,^29^ where bootstrap samples were drawn from each imputed dataset separately, and the estimates were then pooled together. The cost-effectiveness plane was created to visually present 5000 bootstrapped iterations. Furthermore, a cost-effectiveness acceptability curve was drawn to illustrate the likelihood of the intervention being cost-effective across various willingness-to-pay (WTP) thresholds.^30^ All analyses were performed with Stata version 16 for Windows (StataCorp, College Station, Texas) based on an intention-to-treat approach.

### Sensitivity analysis

To ensure the robustness of the primary analysis results, we performed a series of sensitivity analyses. Initially, a cost–utility analysis (CUA) was performed with complete cases to examine the influence of missing data. Subsequently, another CUA was carried out, focusing on the perspective of the UK National Health Service (NHS) and personal social services (PSS) to evaluate the economic impact solely on the NHS. Following that, a CUA was executed from an NHS/PSS and education perspective to consider the combined economic impact on the NHS and education systems. This was done in recognition of the responsibilities held by both NHS organisations and schools in supporting children with autism, as outlined in the Special Educational Needs and Disability (SEND) Code of Practice 2015.^31^ Finally, considering that training costs may be viewed as one-time expenses, a CUA was conducted from the societal perspective, excluding training costs. This aimed to evaluate the impact if the intervention were to be provided over an extended period, thereby eliminating the need for continuous training.

### Funding, ethics and consent statements

This study was funded by the National Institute for Health and Care Research Health Technology Assessment (HTA) programme (grant number 16/111/91), and the clinical trial registration number is ISRCTN11634810. Approval from the North East – York Research Ethics Committee (approval number 19/NE/0237) was secured. Informed consent in writing was acquired from a parent or person with parental responsibility for each child. The study tool into account to the child's willingness to participate, and those who were not willing were not included. The health economics analysis plan was signed off before analysis, and a copy of the plan is held within the ASSSIST-2 Trial Master File at York Trials Unit, University of York, available for inspection upon reasonable request.

### Patient and public involvement

Patient and public involvement is detailed elsewhere.^12^

## Results

### Participants

A total of 295 children were screened from 98 schools, and 249 children from 87 schools had baseline assessment and were randomised (129 children from 44 schools were assigned to the Social Stories arm, and 120 children from 43 schools were allocated to the usual care arm). This configuration serves as the base case. Among the participants, 112 children (45.0%) had complete utility and cost data at baseline and the 6-month follow-up, forming the complete case. In the primary analysis, a total of 29.7% of costs or utilities were initially missing and subsequently imputed.

Table 1 shows the baseline characteristics of the participating children. It reveals that three-quarters of children in both the Social Stories and usual care arms were male. This aligns with the demographic distribution of school-age children with autism in the UK.^32^ More than 80% in both arms fell within the primary school age range, spanning from 7 to 11 years. Minimal differences were observed in parent SRS-2 and EQ-5D-Y-3L proxy utility scores at the baseline across the arms. In summary, the baseline characteristics demonstrate consistency among arms and different samples (base case and complete case).

**Table 1:** Key characteristics at baseline by trial arm

Baseline characteristics	Base case (*n* = 249)		Complete case (*n* = 112)	
	Social Stories (*n* = 129)	Usual care (*n* = 120)	Social Stories (*n* = 58)	Usual care (*n* = 54)
Gender, *n* (%)				
Male	95 (73.6%)	90 (75.0%)	40 (69.0%)	36 (66.7%)
Age (years), *n* (%)				
4–6	22 (17.1%)	24 (20.0%)	11 (19.0%)	10 (18.5%)
7–11	107 (82.9%)	96 (80.0%)	47 (81.0%)	44 (81.5%)
Mean (s.d.)	8.5 (1.7)	8.6 (1.8)	8.3 (1.7)	8.6 (1.7)
Parent SRS-2 scores				
Mean (s.d.)	82.0 (8.4)	82.5 (8.1)	80.6 (9.9)	82.6 (8.0)
SEN status, *n* (%)				
Non-SEN	116 (89.9%)	111 (92.5%)	53 (91.4%)	50 (92.6%)
SEN	13 (10.1%)	9 (7.5%)	5 (8.6%)	4 (7.4%)
Number of participants, *n* (%)				
≤5	80 (62.0%)	70 (58.3%)	31 (53.5%)	32 (59.3%)
>5	49 (38.0%)	50 (41.7%)	27 (46.5%)	22 (40.7%)
EQ-5D-Y-3L proxy utility score at baseline				
Mean (s.d.)	0.75 (0.20)	0.75 (0.20)	0.75 (0.20)	0.74 (0.20)
Societal costs at baseline				
Mean (s.d.)	£3910.9 (9843.0)	£2762.3 (7747.1)	£2190.7 (4478.7)	£2126.0 (3223.7)
Intervention sessions received				
Mean (s.d.)	4.3 (3.3)	−	5.4 (3.1)	−

SRS-2, Social Responsiveness Scale, Second Edition; SEN, special educational needs.

### Health outcome: QALYs

In terms of utility score, both arms showed a slight increase (ranging from 0.01 to 0.04 for utility scores) from baseline to 6-month follow-up, with similar small increments observed in both the base case and complete case samples. Hence, differences between groups in QALYs at 6 months were negligible. Further details are presented in Supplementary File 1, Appendices 4 and 5.

### Cost

The cost per session per child for the intervention was estimated to be £15.22, consisting £12.52 of training costs and £2.70 of delivering intervention costs. The key cost driver of training costs was the trainer fee (71.5%), whereas the main contributors to delivery costs were the costs related to the time interventionists spent on preparation and delivery (58.2%) (refer to Supplementary File 1, Appendix 6).

Regarding service costs, the imputed total costs to the society were £1632.4 (95% CI £1160.3–£2104.5) for Social Stories compared with £1713.6 (95% CI £1211.8–£2215.5) for usual care. For the healthcare-related costs, children in the usual care group had higher costs for child and adolescent mental health services-related community-based services, hospital-based services and non-mental health-related medication. The same trend was observed for school-based costs. Children in the Social Stories group incurred less costs in school-based health services (such as educational psychologist and school nurse visits) and in general support from teachers compared with those in usual care. However, higher costs for school-based intervention services (i.e. one-to-one mentoring/individual work and social communication groups) were observed for Social Stories. Finally, it was observed that Social Stories incurred less costs in parental productivity losses, but slightly higher costs in parental private expenses. It is important to note that certain differences in costs may have been influenced by a few cases with high costs. Specifically, the higher school-based intervention service costs and higher private expenses were driven by two cases each. The analysis retained these high-cost cases because they were deemed plausible. However, caution should be exercised in interpreting the cost differences because of the existence of these high-cost cases. Table 2 presents information on the service use costs broken down by perspective, service type, trial arm and before and after imputation, and details of resource use are shown in Supplementary File 1, Appendix 7.

**Table 2:** Breakdown of the service use costs in six months by trial arm

	Base case		Complete case	
	Social Stories (*n* = 129), £ (s.e.)	Usual care (*n* = 120), £ (s.e.)	Social Stories (*n* = 58), £ (s.e.)	Usual care (*n* = 54), £ (s.e.)
NHS and PSS	252.5 (37.9)	351.8 (64.8)	250.4 (48.3)	379.0 (110.2)
Community-based services				
CAMHS related	17.9 (8.9)	53.7 (20.2)	16.0 (10.1)	43.2 (19.9)
Non-CAMHS related	151.5 (27.8)	162.6 (38,2)	150.5 (35.8)	123.9 (24.3)
Hospital-based services				
Mental health related	1.9 (1.9)	6.4 (3.7)	-	14.2 (8.1)
Non-mental health related	36.5 (11.5)	85.3 (46.8)	33.4 (15.2)	145.9 (101.3)
Medications				
Mental health related	35.0 (10.5)	31.5 (8.3)	41.7 (14.6)	34.7 (10.9)
Non-mental health related	9.8 (3.7)	12.4 (4.45)	8.8 (6.3)	17.0 (7.5)
Education system related	725.3 (167.0)	819.6 (128.8)	635.3 (182.8)	701.2 (156.6)
School-based health services	90.9 (26.5)	253.9 (73.2)	138.8 (46.0)	244.6 (90.2)
Intervention support	497.3 (160.3)	212.6 (60.1)	328.6 (155.0)	230.0 (94.7)
General support	187.1 (41.2)	353.2 (78.6)	167.9 (51.8)	226.7 (69.2)
Private expenses	589.0 (176.3)	425.4 (168.5)	624.6 (254.6)	575.1 (288.6)
Parental productivity loss	80.8 (22.4)	116.8 (34.9)	65.6 (24.4)	116.9 (38.6)
Total costs	1632.4 (237.7)	1713.6 (252.8)	1575.9 (315.6)	1772.2 (411.1)

NHS, National Health Service; PSS, personal social services; CAMHS, Child and Adolescent Mental Health Services.

### CUA (primary analysis)

**Fig. 1:**
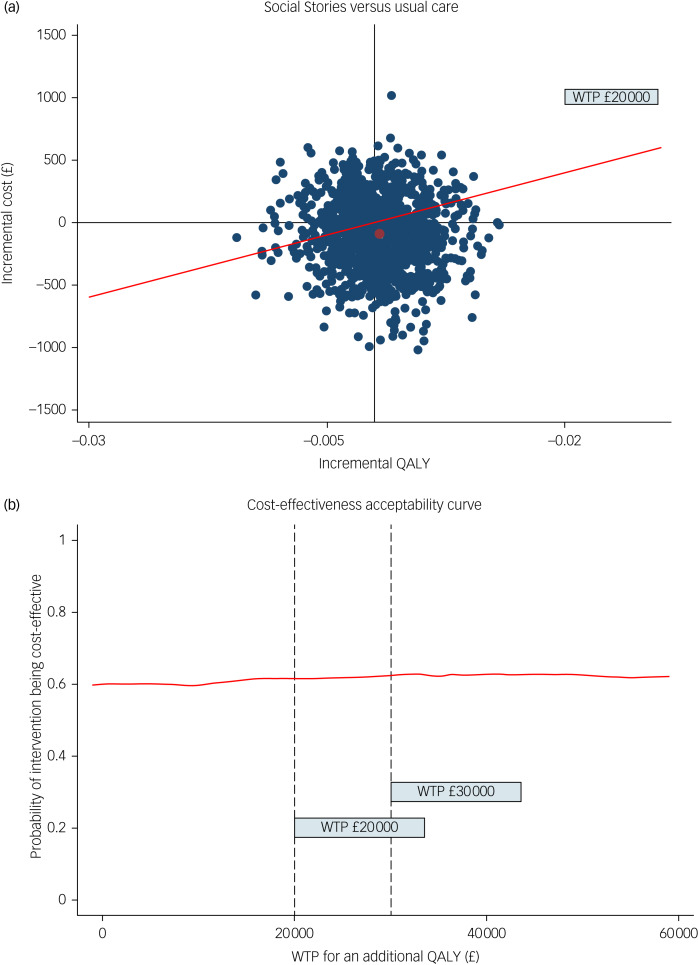
Cost-effectiveness plane and cost-effectiveness acceptability curve. QALY, quality-adjusted life-year; WTP, willingness to pay.

After adjusting for the imbalanced characteristics at baseline, on average, children receiving Social Stories incurred £85.5 (95% CI −£472.6 to £692.8) less costs from the societal perspective (not statistically significant) and maintained similar QALYs (mean incremental difference: 0.001, 95% CI −0.008 to 0.009), compared with those in the usual care group. The 5000 bootstrapped ICER estimates are displayed in Fig. 1(a). It is evident from the illustration that the majority of the simulated estimates fell below the £20 000 per QALY gained threshold line. This implies that if the society is prepared to spend £20 000 for each additional QALY gained, then Social Stories is likely to be the preferred option over usual care, although the incremental cost saving were small and incremental QALYs were similar. The cost-effectiveness acceptability curve in Fig. 1(b) illustrates the likelihood of Social Stories being cost-effective across various WTP thresholds. As shown, if society is willing to spend £20 000 for each QALY gained, the likelihood of Social Stories being a preferred option is 62%, increasing to 63% when the WTP threshold is set at £30 000 per QALY gained.

### Sensitivity analysis

**Fig. 2:**
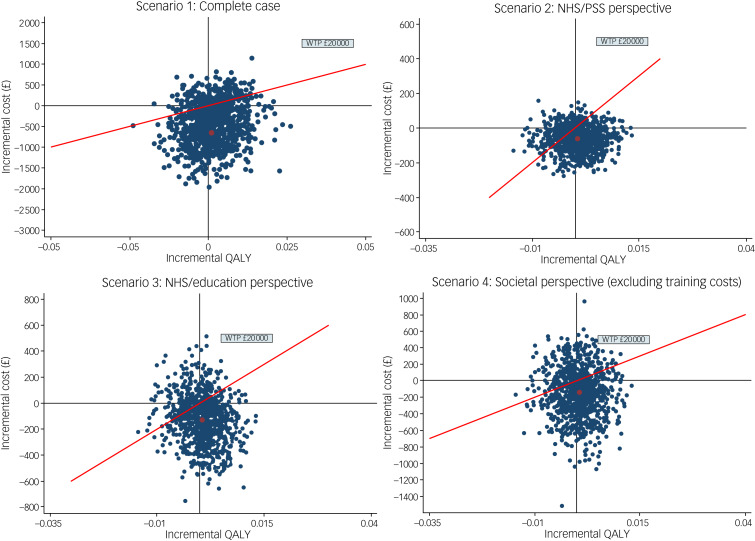
Cost-effectiveness planes of sensitivity analyses. NHS, National Health Service; PSS, personal social services; QALY, quality-adjusted life-year; WTP, willingness to pay.

The outcomes of the sensitivity analyses are visualised in Fig. 2 and detailed in Supplementary File 1, Appendix 8. As depicted, the mean incremental costs and QALYs derived from the complete cases closely align with the base case scenario, resulting in a negative cost per QALY gained. Similar results were also observed from the sensitivity analyses conducted from the perspective of the NHS/PSS (scenario 2), the NHS/PSS and education perspective (scenario 3), and the societal perspective excluding training costs (scenario 4). All of the sensitivity analyses demonstrated that Social Stories is dominant, and a good proportion of the bootstrapped estimates fell below the WTP threshold recommended by National Institute for Health and Care Excellence (£20 000 QALY gained), especially when the NHS/PSS and education perspective was adopted (Scenario 3).

## Discussion

### Principal findings

To the best of our knowledge, this study is the first trial-based investigation to evaluate the cost-effectiveness of Carol Gray's Social Stories in children with autism. Our study indicates that, compared with usual care, Social Stories did not lead to an increase in service use costs to the society, and maintained similar QALY improvements. Furthermore, the possibility of Social Stories being cost-effective is <70%, indicating a high degree of uncertainty regarding the cost-effectiveness of the intervention. Sensitivity analyses, incorporating costs measured from different perspectives, yield consistent findings.

### Implications of study

The results in Table 2 show a small, but not statistically significant reduction in costs across different perspectives. Such reduction was particularly evident in the education sector, including costs such as school-based health services and general support from teachers and/or teaching assistants in school. Although school-based intervention costs in the Social Stories arm were identified as higher, this appears to be associated with a limited number of high-cost cases in this group rather than indicating a widespread increase in costs across the entire arm. The finding suggests that Social Stories may have the potential to reduce the support needed in schools without affecting the usual school-based services/interventions that children with autism receive. Moreover, since the intervention not only provides social information to children, but also establishes a dialogue between teachers, parents and child, it is likely to improve the adult understanding of children's needs. This, in turn, enables proactive and preventive actions that may lead to less demand for higher level of support and associated costs later on. It is noteworthy that, although our results indicated a small to negligible cost reduction, the prospective savings could hold significance for commissioners when assessing resource utilisation on a larger scale and multiplying the impact by the number of children with autism in the UK. Currently, there are around 98 000 children with autism aged 4–11 years in England,^2,33^ and more than 70% of children with autism are educated in mainstream schools.^34^ This implies a potential cost saving to the NHS and education system of around £9 million for the primary-aged children with autism population as a whole. However, caution is warranted in interpreting this relatively positive finding for two reasons. First, the cost reduction was not statistically significant. Second, a comprehensive long-term analysis is necessary to ensure the sustained effectiveness of Social Stories over an extended period beyond the initial 6-month timeframe, and to verify the persistence of the associated cost savings. If the long-term analysis confirms sustained cost savings, exploring potential joint funding arrangements between the NHS and the education sector could be a viable approach to finance the implementation of Social Stories in the UK. This is because NHS organisations and schools share the responsibilities and financing to support children with autism based on the SEND Code of Practice 2015.^31^

On the other hand, the disparity in QALY improvements between the two arms was minimal (Supplementary File 1, Appendix 4). Even after considering uncertainty and imbalanced baseline data, the difference in QALYs remains minimal (≦0.001 QALYs), suggesting that Social Stories do not appear to improve the quality of life (measured by EQ-5D-Y-3L proxy) of children with autism. However, using a health-related quality of life instrument more attuned to changes in mental well-being for neurodiverse children and young people could improve future research such as this.

### Strengths and limitations

The study is the first to assess the cost-effectiveness of Social Stories, and the data were collected from a fully powered, randomised controlled trial. Such study designs allow more robust estimates to be generated. The adopted multi-perspective approach was another strength. The evaluation accounted for the costs from various perspectives (NHS/PSS, NHS/PSS/education and societal perspectives), making the evaluation results useful to a broad spectrum of stakeholders, including health policy makers, education sectors and parents/guardians of children with autism. Furthermore, the impact of missing data was explored through sensitivity analysis. This approach not only provides reassurance with regards to the robustness of our findings, but also aids policy makers across diverse sectors in making well-informed decisions.

However, the economic evaluation had certain limitations. Chief among them was the issue of children who were unable to receive Social Stories or experienced variance in the frequency of Social Stories delivery within the 4-week time period because of the COVID-19 pandemic. This aspect raises concerns as it has the potential to introduce bias into the study results and underestimate the potential cost saving and QALY improvement. However, since the disruption occurred to both arms, it is expected that the impact on our results is limited. Also, there was a non-negligible amount of missingness in the primary analysis. This amount of missing data may introduce bias and limit the precision of the conclusions. Although the presence of these missing data does introduce additional uncertainty with regards to the substantive conclusion, the apparent insensitivity of the results to different missing-at-random assumptions provides some reassurance. Further, a few high-cost cases were observed, and they may affect the interpretation of certain cost comparison outcomes (see Results). However, these high-cost cases are unlikely to affect the direction of the economic results about Social Stories. This is because these high-cost cases are in the Social Stories arm. Also, although the brevity of EQ-5D-Y was a necessity during the challenging study period (the COVID-19 pandemic), it may not be the optimal instrument for children and young people with autism because of concerns regarding the sensitivity of the EQ-5D to mental health conditions. Although both the EQ-5D and EQ-5D-Y have shown that they can be reliably and validly applied to a broad category of neurodevelopmental differences,^16–18^ future research exploring the utilisation of alternative or supplementary instruments, such as the Pediatric Quality-of-Life Inventory (PedsQL™), is recommended. Finally, this economic evaluation assesses the cost-effectiveness of Social Stories over the short term (6 month), leaving the long-term effects unknown. Although not within the current study's scope, future research would benefit from conducting a model-based economic evaluation. This approach would enable the measurement of long-term cost-effectiveness and the evaluation of the impact on children's productivity as they transition into adulthood.

In conclusion, the current study demonstrates no increase in costs in delivering Social Stories to children with autism in mainstream school settings, and it sustains comparable QALYs. This observation holds true in both primary and sensitivity analyses. The results will be relevant to policy makers, healthcare providers, education sectors and the parents/guardians of children with autism.

## Supporting information

Wang et al. supplementary material 1 Wang et al. supplementary material

Wang et al. supplementary material 2 Wang et al. supplementary material

## Data Availability

Data supporting the findings can be obtained on reasonable request from the corresponding author, H.I.W. Requests for access to the ASSSIST-2 data will be reviewed on an individual basis by the Chief Investigator.
