# Supplementary material for: Cost–utility analysis of Social Stories™ for children with autism spectrum disorder in mainstream primary schools: results from a randomised controlled trial
Source: BJPsych Open. 2024 Jun 3;10(4):e123. doi: 10.1192/bjo.2024.47 (PMC11363088; doi:10.1192/bjo.2024.47)
Supplement: Wang et al. supplementary material 1 — Wang et al. supplementary material [file S2056472424000474sup001.pdf]

Appendix 1 Inclusion and exclusion criteria

Inclusion criteria

- The child was aged 4-11 years at the time of recruitment.
- The child attended a participating primary or SEN school within Yorkshire and the Humber.
- The child has a clinical diagnosis of Autism Spectrum Condition as confirmed by the parent/carer during a screening call and had daily challenging behaviour as confirmed during a screening call with the child’s teacher.
- Parents/guardians of the child were able to self-complete the English language outcome measures (with assistance if necessary).

Exclusion criteria

- The school had used Social Stories™ for any pupil in the current or preceding school term.
- The child or interventionist teacher had taken part in the previous Social Stories™ feasibility study (ASSSIST). Schools that have taken part were not excluded.

Appendix 2 Flow diagram

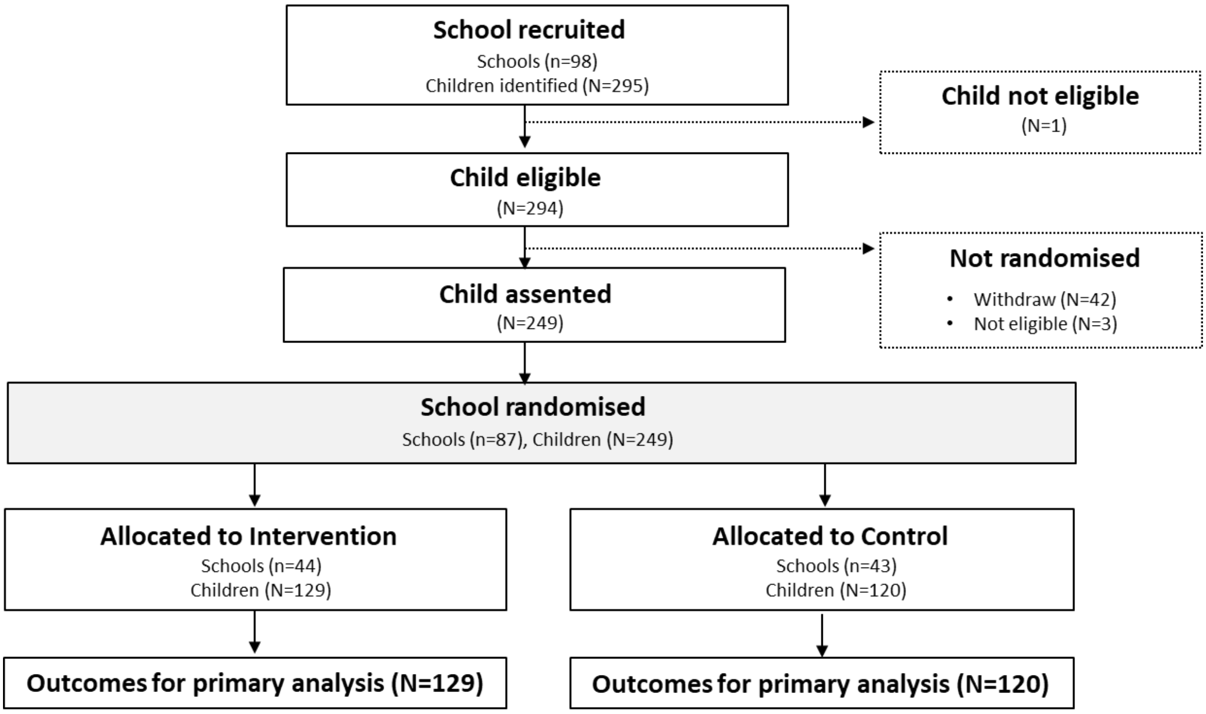

Appendix 3 List of unit costs

General health community services

Item	Unit cost (£)			Source
	At home**	At clinic / surgery	Via phone / email	
General practitioner (GP)	£45.9	£39.2	£37.6	PSSRU 2019 (chapter 10.3, 10.4)
Community nurse*	£55.7	£49.0	£49.0	PSSRU 2019 (chapter 6.1)
Community Paediatrics	£161.7	£155.0	£155.0	PSSRU 2019 (chapter 6.1)
Social care worker*	£28.7	£22.0	£22.0	PSSRU 2019 (chapter 11.2)
Home care worker*	£20.7	£14.0	£14.0	PSSRU 2019 (chapter 11.5)
Family support worker***	£23.2	£16.5	£16.5	PSSRU 2018 (chapter 6.9)
Helpline****	-	-	£6.0	PSSRU 2019 (chapter 10.5)
Occupational therapist	£151.7	£145.0	£145.0	PSSRU 2019 (chapter 6.1)
Physiotherapist	£87.7	£81.0	£81.0	PSSRU 2019 (chapter 6.1)
Speech and Language Therapist	£89.7	£83.0	£83.0	PSSRU 2019 (chapter 6.1)
Dentist*	£73.2	£66.5	£66.5	PSSRU 2019 (chapter 10.6)
Dietician	£92.7	£86.0	£86.0	PSSRU 2019 (chapter 7.1)

*Assume the duration of visit is 30 minutes
**Including the average travel time of 12 minutes per visit (PSSRU 2015).
***Unit cost was inflated to 2019 prices based on originally reported values from PSSUR 2018/19 (Curtis and Burns 2018)
****Assume the cost is the same as telephone triage led by nurse

Mental health related, community based services

Item	Unit cost (£)	source
Child psychiatrist	£133.0	PSSRU 2019 (chapter 9)
Child psychotherapist	£94.0	PSSRU 2019 (chapter 6.10)
Child psychologist or clinical psychologist*	£109.0	PSSRU 2019 (chapter 9)
Mental health nurse or CAMHS therapist*	£60.0	PSSRU 2019 (chapter 10.1)

*Assume 1 hour per session

Hospital-based services

Item	Unit cost (£)	Source
Accident and Emergency	£142.4	National Cost Collection 2019/20 (service code: T01NA)
NHS walk-in centre	£34.7	National Cost Collection 2019/20 (service code: T04NA)
Urgent Care Centre	£34.7	National Cost Collection 2019/20 (service code: T04NA)
Outpatient visit - Paediatrics	£232.0	National Cost Collection 2019/20 (service code: 420)
Outpatient visit - Ear Nose and Throat	£124.0	National Cost Collection 2019/20 (service code: 215)
Outpatient visit - Allergy	£247.0	National Cost Collection 2019/20 (service code: 255)
Outpatient visit - Epilepsy	£222.0	National Cost Collection 2019/20 (service code: 223)
Outpatient visit - Paediatrics	£232.0	National Cost Collection 2019/20 (service code: 420)
Outpatient visit - Dental Medicine	£171.0	National Cost Collection 2019/20 (service code: 450)

Outpatient visit - Dermatology	£170.0	National Cost Collection 2019/20 (service code: 257)
Outpatient visit - Cardiology	£142.0	National Cost Collection 2019/20 (service code: 320)
Outpatient visit - Occupational Therapy	£73.0	National Cost Collection 2019/20 (service code: 651)
Outpatient visit - Podiatry	£62.0	National Cost Collection 2019/20 (service code: 653)
Outpatient visit - Ophthalmology	£103.0	National Cost Collection 2019/20 (service code: 216)
Outpatient visit - Psychiatry	£361.0	National Cost Collection 2019/20 (service code: 711)
Day case - bladder condition	£1,031.0	National Cost Collection 2019/20 (HRG code:PX08C)
Day case - Non-Surgical Ophthalmology	£558.0	National Cost Collection 2019/20 (HRG code:PP64B)
Day case – Ear condition	£1,021.0	National Cost Collection 2019/20 (HRG code:PP64B)
Day case – Bone condition	£771.0	National Cost Collection 2019/20 (HRG code:PX05C)
Day case - Dental procedure	£683.0	National Cost Collection 2019/20 (HRG code:CD03B)
Inpatient stay - arm fracture	£5,281.0	National Cost Collection 2019/20 (HRG code: HE51C)

Medications

Item	Chemical name	Dosage	Unit cost (£)	Source
Melatonin_Tab 2mg	Melatonin	2mg tablet (once daily)	£1.33 per quantity	PCA 2019 (0401010ADAABKBK)
Melatonin_ 2mg / 5ml oral solution	Melatonin	2mg / 5ml (once daily)	£0.17 per quantity	PCA2019 (0401010ADAABYBY)
Clenil Modulite_Inha 50mcg*	Beclometasone Dipropionate	50mcg (2 puffs daily)	£3.70 per quantity	PCA 2019 (0302000C0BPAABE)
Salbutamol_Inha 100mcg*	Salbutamol	2 puffs (as required)	£1.70 per quantity	PCA 2019 (0301011R0AAAAAA)
Movicol_Paed Pdr Sach	Macrogol 3350	6.9g sachet (2 sachets a day)	£0.30 per quantity	PCA 2018 (0106040M0BBAIAB)
Equasym XL_tab_ 30mg	Methylphenidate hydrochloride	30mg tablet (once daily)	£1.17 per quantity	PCA 2019 (0404000M0BCAFAV)
Methylphenidate_tab 10mg	Methylphenidate hydrochloride	10mg tablet (once daily)	£0.12 per quantity	PCA 2019 (0404000M0AAAAAA)
Dulcolax Pico_liquid 5mg/5ml	Sodium picosulfate	5mg/5ml (once daily)	£0.02 per quantity	PCA 2019 (0106020P0BDACAB)
Cetirizine_tab 10mg	Cetirizine Hydrochloride	10mg tablet (once daily)	£0.03 per quantity	PCA 2019 (0304010I0AAAAAA)
Lactulose_oral solution 10g/15ml	Lactulose	10g/15ml (once daily)	£0.25 per quantity	PCA 2019 (0106040G0AAAGAG)
Montelukast_tab 5mg	Montelukast	5mg tablet (once daily)	£0.05 per quantity	PCA 2019 (0303020G0AAAAAA)

*Assume each inhaler contains 200 puffs

Note: The medication costs were based on the Prescription Cost Analysis 2019. Due to the significant amount of information, further details are available upon request.

School-based health services

Item	Unit cost (£)	Source*
Educational psychologist	£23.6/hour	NJC green book 2019 (SCP 43)
Education welfare officer	£12.4/hour	NJC green book 2019 (SCP 17)
School or college nurse	£12.4/hour	NJC green book 2019 (SCP 17)

*Based on the average Spinal Column Point (SCP) from the National Joint Council (NJC) for Local Government Services - green book 2019

(https://neu.org.uk/support-staff). Hourly rate was calculated by dividing annual salary by 52.143 weeks (which is 365 days divided by 7) and then divided by 37 hours (the standard working week in the National Agreement 'Green Book')

Education services/support

Item	Unit cost (£)	Source*
Teacher	£16.6/hour	NJC green book 2019 (SCP 29)
TA (teaching assistant)	£9.9/hour	NJC green book 2019 (SCP 6)
HLTA (high level teaching assistant)	£11.2/hour	NJC green book 2019 (SCP 12)
SENCO (special educational needs coordinator)	£19.6/hour	NJC green book 2019 (SCP 36)
SEN (special educational needs) assistant	£11.9/hour	NJC green book 2019 (SCP 15)
LM (learning mentor)	£11.6/hour	NJC green book 2019 (SCP 14)
ELSA (emotional literacy support assistant head)	£10.1/hour	NJC green book 2019 (SCP 7)
Literacy support assistant	£11.0/hour	NJC green book 2019 (SCP 11)
STA (specialist teacher advisor)	£14.5/hour	NJC green book 2019 (SCP 24)
Other	£9.9/hour	Assume the same as TA

*Based on the average Spinal Column Point (SCP) from the National Joint Council (NJC) for Local Government Services - green book 2019 (https://neu.org.uk/support-staff). Hourly rate was calculated by dividing annual salary by 52.143 weeks (which is 365 days divided by 7) and then divided by 37 hours (the standard working week in the National Agreement 'Green Book')

Private expenses

**Item**	**Unit cost (£)**	**Source**
Privately paid mental health services	£360.0/session	https://www.psychiatry-uk.com/fees/
After school club	£57.4/week	Childcare survey 2019
Holiday club	£133.4/week	Childcare survey 2019
Day care	£158.6/day	PSSRU 2019 (chapter 6.11)
Childcare - home support	£25.8/hour	PSSRU 2019 (chapter 6.11)
Personal assistant (PA)	£8.2/hour	National minimal wage 2019
Swimming club	£4.58 / session	2019 State of the UK swimming industry report https://www.leisuredb.com/publications/

Productivity cost

**Item**	**Unit cost (£)**	**Source**
Average weekly earnings (AWE) of employed people in the UK	£585/week	Office for National Statistics 2019

Appendix 4 Utility scores and QALYs at baseline and month 6 by trial arm

	Base case		Complete case	
**Time point**	**Social Stories™ (n=129), mean (95% CI)**	**Usual care (n=120) mean (95% CI)**	**Social Stories™ (n=58), mean (95% CI)**	**Usual care (n=54) mean (95% CI)**
Utility score				
Baseline	0.75 (0.72, 0.79)	0.75 (0.71, 0.78)	0.75 (0.70, 0.80)	0.74 (0.68, 0.79)
Month 6	0.78 (0.75, 0.81)	0.76 (0.74, 0.81)	0.79 (0.75, 0.83)	0.78 (0.74, 0.83)
Total QALYs	0.38 (0.37, 0.40)	0.38 (0.37, 0.40)	0.38 (0.36, 0.41)	0.38 (0.36, 0.40)

Appendix 5 EQ-5D-Y responses by trial arm by data collection time point

Social Stories (n=58)	Baseline			Month 6		
	Level 1<br>n (%)	Level 2<br>n (%)	Level 3<br>n (%)	Level 1<br>n (%)	Level 2<br>n (%)	Level 3<br>n (%)
Mobility	39 (67.2)	15 (25.9)	4 (6.9)	43 (74.1)	13 (22.4)	2 (3.5)
Self-care	14 (24.1)	27 (46.6)	17 (29.3)	16 (27.6)	29 (50.0)	13 (22.4)
Usual activity	25 (43.1)	26 (44.8)	7 (12.1)	26 (44.8)	28 (48.3)	4 (6.9)
Pain/discomfort	41 (70.7)	15 (25.9)	12 (3.4)	42 (72.4)	15 (25.9)	1 (1.7)
Anxiety/depression	20 (34.5)	33 (56.9)	5 (8.6)	22 (37.9)	32 (55.2)	4 (6.9)
**Usual care (n=54)**	**Baseline**			**Month 6**		
	**Level 1**<br>**n (%)**	**Level 2**<br>**n (%)**	**Level 3**<br>**n (%)**	**Level 1**<br>**n (%)**	**Level 2**<br>**n (%)**	**Level 3**<br>**n (%)**
Mobility	38 (70.4)	14 (25.9)	2 (3.7)	39 (72.2)	15 (27.8)	-
Self-care	12 (22.2)	29 (53.7)	13 (24.1)	14 (25.9)	29 (53.7)	11 (20.4)
Usual activity	19 (35.2)	31 (57.4)	4 (7.4)	19 (35.2)	29 (53.7)	6 (11.1)
Pain/discomfort	39 (72.2)	13 (24.1)	2 (3.7)	38 (70.4)	15 (27.8)	1 (1.8)
Anxiety/depression	22 (40.7)	19 (35.2)	13 (24.1)	24 (44.4)	25 (46.3)	5 (9.3)

Appendix 6 Breakdown of the intervention costs

	Total cost (£)	Cost per session per child (£)
Training costs		
Trainer fee (staff time)		
Preparation	£922.91	£1.65
Training	£4,088.73	£7.30
Trainer's travel costs	£1,080.96	£1.93
Consumable costs	£918.05	£1.64
Total	£7,010.65	£12.52
Intervention delivery costs		
Intervention (staff time)		
Therapy session (preparation and delivery)	£880.33	£1.57
Additional work/help from other staff	£97.13	£0.17
Consumables	£313.60	£0.56
Supervision*	£220.50	£0.39
Total	£1511.56	£2.70

*The calculation method and unit cost were based on Wiles et al. study: Wiles N, Thomas L, Abel A et al. Clinical effectiveness and cost-effectiveness of cognitive behavioural therapy as an adjunct to pharmacotherapy for treatment-resistant depression in primary care: the CoBalT randomised controlled trial. Health Technol Assess. 2014 May;18(31):1-167.

Appendix 7 Breakdown of the service use by trial arm

		Baseline		Month 6	
	Unit	Social Stories™, N=58, Mean (sd)	Usual care, N=54, Mean (sd)	Social Stories™, N=58, Mean (sd)	Usual care, N=54, Mean (sd)
NHS and PSS					
Community-based services					
CAMHS related	Session	0.55 (2.26)	0.61 (2.02)	0.22 (0.88)	0.44 (1.34)
Non-CAMHS related					
GP	Appointment	0.98 (2.27)	0.67 (1.17)	0.58 (1.43)	0.46 (0.88)
Allied health professionals	Appointment	1.81 (3.15)	2.26 (3.70)	1.24 (2.79)	1.44 (3.06)
Social care service	Appointment	1.97 (9.13)	0.52 (2.00)	0.45 (1.57)	0.13 (0.67)
Hospital-based services / acute services					
Emergency services	Visit	0.22 (0.80)	0.17 (0.38)	0.16 (0.45)	0.20 (0.59)
Inpatient stay					
Mental health related	Night	-	-	-	-
Non-mental health related	Night	-	-	-	0.02 (0.14)
Outpatient visit / day case					
Mental health related	Visit	0.07 (0.26)	0.07 (0.26)	-	0.09 (0.35)
Non-mental health related	Visit	0.20 (0.44)	0.20 (0.49)	0.10 (0.31)	0.15 (0.45)
Medication					
Mental health related	Type	0.31 (0.65)	0.31 (0.72)	0.21 (0.45)	0.26 (0.56)
Non-mental health related	Type	0.38 (0.81)	0.50 (0.84)	0.22 (0.46)	0.22 (0.50)
Education system related					

School-based health services	Hour	12.86 (35.45)	5.78 (13.94)	9.58 (23.7)	15.43 (44.24)
Intervention support*	Hour	74.17 (156.35)	71.91 (83.56)	48.36 (107.54)	55.24 (95.83)
General support*	Hour	1.78 (1.62)	2.28 (2.16)	2.19 (1.74)	2.96 (2.60)
Private expanses – out of pocket					
Privately paid consultation	Session	-	-	0.21 (1.58)	0.07 (0.54)
Childcare	Session	5.84 (18.68)	3.02 (11.70)	7.24 (25.24)	6.91 (28.40)
Club	Session	3.76 (10.47)	7.33 (17.62)	1.55 (5.51)	4.72 (12.85)
Productivity					
Parental productivity	Day	0.86 (2.16)	0.81 (1.78)	0.98 (2.90)	1.70 (3.53)

CAMHS: Child and Adolescent Mental Health Services, including child psychiatrist, child psychotherapist, child psychologist, clinical psychologist, mental health nurse, family therapist, and Primary mental health worker (PMHW)

Allied health professionals for the community-based services included community nurse, community paediatrician, occupational therapist, physiotherapist, and Speech and Language therapist

Social care services included social care worker, home care worker, family support worker, drug and alcohol support worker, and Helpline (e.g. Samaritans)

Club included after-school clubs, religious clubs, sport clubs and special clubs for autism children

*based on the teacher-reported questionnaires

Appendix 8 Results of sensitivity analyses

Social Stories™ vs. usual care	Incremental costs (£), (95% CI)	Incremental QALYs (95% CI)	ICER (£/QALY gained), (95% CI)
**Scenario 1**: Complete case analysis from the societal perspective	-400.2 (-1331.0, 463.3)	0.001 (-0.014, 0.012)	Dominant
**Scenario 2**: CEA from the NHS/PSS perspective	-60.5 (-214.7, 66.7)	0.001 (-0.008, 0.009)	Dominant
**Scenario 3**: CEA from the NHS/PSS and education perspective	-128.9 (-485.4, 236.6)	0.001 (-0.008, 0.009)	Dominant
**Scenario 4:** CEA from the societal perspective (excluding training costs)	-134.0 (-794.7, 284.6)	0.001 (-0.007, 0.010)	Dominant
