# Supplementary material for: Cost–utility analysis of Social Stories™ for children with autism spectrum disorder in mainstream primary schools: results from a randomised controlled trial
Source: BJPsych Open. 2024 Jun 3;10(4):e123. doi: 10.1192/bjo.2024.47 (PMC11363088; doi:10.1192/bjo.2024.47)
Supplement: Wang et al. supplementary material 2 — Wang et al. supplementary material [file S2056472424000474sup002.pdf]

Resource use questionnaire – parent/guardian

We would like to understand your child's use of services, excluding any Social Stories™.
This will help us understand how much support your child gets in different places.

a. *If you are not sure about the service name, then please select the one you think is the closest match.*

b. *All appointments or visits should be counted only ONCE.*

General health and community services

Has your child accessed any of the following in the last 6 months? *(please cross all that apply)*

☒ Service	Number of appointments attended: *At home*	*At clinic/ surgery*	*Via phone/ email*
☐ General Practitioner (GP)			
☐ Community Nurse			
☐ Community Paediatrician			
☐ Social care worker			
☐ Home care worker			
☐ Family support worker			
☐ Drug and alcohol support worker			
☐ Helpline (e.g. Samaritans)			
☐ Occupational therapist			
☐ Physiotherapist			
☐ Speech and Language Therapist			
☐ Youth worker			
☐ Other (please specify):			

Has your child accessed any of the following respite and sharing care community-based services in the last 6 months? *(please cross all that apply)*

☒	
☐ Overnight stay in respite centre / Local Authority house	nights
☐ Overnight stay with another family / sharing care / PACT stay	nights
☐ Day stay with another family / sharing care / PACT support	days

Education services

Has your child accessed any of the following educational services in the last 6 months?
(please cross all that apply)

☒	Number of hours per week *At home*	Number of hours per week *At clinic/surgery*
☐ Educational psychologist		
☐ Education welfare officer		
☐ School nurse		
☐ Specialist teacher advisor (e.g. for autism)		

Mental health services

Has your child had any appointments with the Child and Adolescent Mental Health Services (CAMHS) in the last 6 months? *(Please do not count visits or appointments included in the previous tables.) (please cross all that apply)*

☒	Number of sessions
☐ Child psychiatrist	
☐ Child psychotherapist	
☐ Child psychologist or clinical psychologist	
☐ Mental health nurse or CAMHS therapist	
☐ Family therapist	
☐ Wellbeing worker	
☐ Other (please specify):	

Has your child received any other mental health service in the last 6 months? *(Please do not count visits or appointments included in the previous tables.) (please cross all that apply)*

☒	Number of sessions
☐ Charity support worker	
☐ GP counselling	
☐ School or other counsellor	
☐ Privately paid mental health service *(please specify)*:	
☐ Other *(please specify)*:	

Other services

Has your child accessed any other services in the last 6 months? *(please cross all that apply)*

☒		Number of sessions
☐	Paid childcare (half day = 1 session)	☐☐☐
☐	Other (e.g. Saturday clubs) *(please specify)*:	☐☐☐

Hospital-based services

Has your child accessed any of the following urgent hospital services for any health issue, in the last 6 months? *(please cross all that apply)*

☒		Number of visits	*Was the visit related to autism or any treatment for autism?*
☐	Accident and Emergency (excluding urgent care/ minor injuries unit)	☐☐☐	☐ Yes ☐ No ☐ Unsure
☐	NHS walk-in centre (specify whether 111 call or attendance at centre)	☐☐☐	☐ Yes ☐ No ☐ Unsure
☐	Urgent care centre/ minor injuries unit (excluding A&E attendance or walk-in-centre visits)	☐☐☐	☐ Yes ☐ No ☐ Unsure
☐	Other *(please specify)*:	☐☐☐	☐ Yes ☐ No ☐ Unsure

Has your child had any non-urgent visits to hospital for any health issue, in the last 6 months?

	Details	Day case or overnight stay?	Total nights *(if overnight stay)*	***Was the visit related to autism or any treatment for autism?***
1.		☐ Outpatients<br>☐ Day case<br>☐ Overnight stay	☐☐☐	☐ Yes<br>☐ No<br>☐ Unsure
2.		☐ Outpatients<br>☐ Day case<br>☐ Overnight stay	☐☐☐	☐ Yes<br>☐ No<br>☐ Unsure
3.		☐ Outpatients<br>☐ Day case<br>☐ Overnight stay	☐☐☐	☐ Yes<br>☐ No<br>☐ Unsure
4.		☐ Outpatients<br>☐ Day case<br>☐ Overnight stay	☐☐☐	☐ Yes<br>☐ No<br>☐ Unsure
5.		☐ Outpatients<br>☐ Day case<br>☐ Overnight stay	☐☐☐	☐ Yes<br>☐ No<br>☐ Unsure

Has your child taken any regular medications in the last 6 months (not including painkillers e.g. paracetamol)? ☐ Yes ☐ No *(If 'Yes', please complete the table below)*

	Medication start	Medication finish
1. **Medication name** ☐<br>**Dose and unit** *(e.g. 200mg, 1g etc.)* ☐<br>**Frequency** *(e.g., daily, twice daily, as required)* ☐	Started >6 months ago ☐<br>Start date (if started <6 months ago) *ddmmyyyy*<br>☐☐ / ☐☐ / ☐☐☐☐	Ongoing ☐<br>Stop date (if not ongoing) *ddmmyyyy*<br>☐☐ / ☐☐ / ☐☐☐☐
2. **Medication name** ☐<br>**Dose and unit** *(e.g. 200mg, 1g etc.)* ☐<br>**Frequency** *(e.g., daily, twice daily, as required)* ☐	Started >6 months ago ☐<br>Start date (if started <6 months ago) *ddmmyyyy*<br>☐☐ / ☐☐ / ☐☐☐☐	Ongoing ☐<br>Stop date (if not ongoing) *ddmmyyyy*<br>☐☐ / ☐☐ / ☐☐☐☐
3. **Medication name** ☐<br>**Dose and unit** *(e.g. 200mg, 1g etc.)* ☐<br>**Frequency** *(e.g., daily, twice daily, as required)* ☐	Started >6 months ago ☐<br>Start date (if started <6 months ago) *ddmmyyyy*<br>☐☐ / ☐☐ / ☐☐☐☐	Ongoing ☐<br>Stop date (if not ongoing) *ddmmyyyy*<br>☐☐ / ☐☐ / ☐☐☐☐
4. **Medication name** ☐<br>**Dose and unit** *(e.g. 200mg, 1g etc.)* ☐<br>**Frequency** *(e.g., daily, twice daily, as required)* ☐	Started >6 months ago ☐<br>Start date (if started <6 months ago) *ddmmyyyy*<br>☐☐ / ☐☐ / ☐☐☐☐	Ongoing ☐<br>Stop date (if not ongoing) *ddmmyyyy*<br>☐☐ / ☐☐ / ☐☐☐☐
5. **Medication name** ☐<br>**Dose and unit** *(e.g. 200mg, 1g etc.)* ☐<br>**Frequency** *(e.g., daily, twice daily, as required)* ☐	Started >6 months ago ☐<br>Start date (if started <6 months ago) *ddmmyyyy*<br>☐☐ / ☐☐ / ☐☐☐☐	Ongoing ☐<br>Stop date (if not ongoing) *ddmmyyyy*<br>☐☐ / ☐☐ / ☐☐☐☐

Has your child been absent from school (excluding school holidays) in the last 6 months?
(please cross one box only)

☐ Yes ☐ No

How many days were missed? ☐☐☐

Have you been in employment/education/training in the last 6 months?
(please cross all that apply)

☒	Job title *(if applicable)*	Full/part time	Have you missed any days to care for your child?	Days missed
☐ Paid employment		☐ FT ☐ PT	☐ Yes ☐ No	☐☐☐
☐ Unpaid employment		☐ FT ☐ PT	☐ Yes ☐ No	☐☐☐
☐ Education/training		☐ FT ☐ PT	☐ Yes ☐ No	☐☐☐

Has your partner been in employment/education/training in the last 6 months?
(please cross all that apply)

☒	Job title *(if applicable)*	Full/part time	Have you missed any days to care for your child?	Days missed
☐ Paid employment		☐ FT ☐ PT	☐ Yes ☐ No	☐☐☐
☐ Unpaid employment		☐ FT ☐ PT	☐ Yes ☐ No	☐☐☐
☐ Education/training		☐ FT ☐ PT	☐ Yes ☐ No	☐☐☐

Have you accompanied your child to health or other services in the last 6 months?
(please cross one box only)

☐ Yes ☐ No

Which services were accessed?

Resource use questionnaire – teacher

Has the child received any of the following interventions/support in the last 6 months?
(please cross all that apply)

- ☐ Applied Behavioural Analysis (ABA)
- ☐ Art therapy
- ☐ Discrete trial training
- ☐ Drama therapy
- ☐ ELSA (or equivalent) sessions
- ☐ Functional communication training
- ☐ Intensive interaction
- ☐ LEGO®-based therapy
- ☐ Music therapy
- ☐ Picture Exchange Communication Systems (PECS)
- ☐ Relationship Development Intervention (RDI)
- ☐ Responsive teaching
- ☐ Social Communication groups (e.g. Time to talk)
- ☐ Social skills work group (more then usual care as usual groups)
- ☐ Social Stories™
- ☐ Social Use of Language Programming (SULP)
- ☐ Social-Communication, Emotional Regulation and Transactional Support (SCERTS model)
- ☐ Son-Rise program
- ☐ Structure, Positive (approaches and expectations), Empathy, Low arousal, Links (SPELL approach)
- ☐ Theory of mind training
- ☐ Treatment and Education of Autistic and Communication-Handicapped Children (TEACCH)
- ☐ Verbal behaviour approach
- ☐ Video modelling
- ☐ Visual schedules
- ☐ 1:1 mentoring/ individual work
- ☐ Other (please specify):

For each intervention/support crossed above, please complete a row per provider in the table below:

	Intervention / support	Provided by*	Number of sessions in last 6 months	Average session duration *(mins)*	Are you aware of any associated hospital admissions?
1.				mins	☐ Yes ☐ No
2.				mins	☐ Yes ☐ No
3.				mins	☐ Yes ☐ No
4.				mins	☐ Yes ☐ No
5.				mins	☐ Yes ☐ No
6.				mins	☐ Yes ☐ No

* T	Teacher/educator	ELSA	Emotional Literacy Support Assistant Head
TA	Teaching assistant	HT	Head teacher / senior member of staff
HLTA	Higher Level Teaching Assistant	O	Other (specify above)
SENCO	Special Educational Needs Co-ordinator	STA	Specialist teacher advisor
LM	Learning Mentor	EP	Educational Psychologist

In the last 6 months, are you aware of the child being admitted to hospital due to any of the interventions listed on page 5?

Admission details	Date of admission (dd/mm/yyyy)	Length of stay
1.	day / month / year	nights
2.	day / month / year	nights
3.	day / month / year	nights

How many children are usually in the child's class (including the child)? ☐☐ children

Who has been involved in supporting the child in class in the last 6 months?
(only include individual/1:1 assistance in usual classroom lessons) please cross all that apply and provide the additional information.

Support provided by	Number of individuals providing support	Whole Time Equivalent (WTE)	OR	Hours with the child in a week
☐ Teacher / Educator	☐☐	☐.☐☐ WTE		☐☐ hrs ☐☐ mins
☐ Teaching Assistant	☐☐	☐.☐☐ WTE		☐☐ hrs ☐☐ mins
☐ Other *(specify)*	☐☐	☐.☐☐ WTE		☐☐ hrs ☐☐ mins
☐ Other *(specify)*	☐☐	☐.☐☐ WTE		☐☐ hrs ☐☐ mins
☐ Other *(specify)*	☐☐	☐.☐☐ WTE		☐☐ hrs ☐☐ mins
☐ Other *(specify)*	☐☐	☐.☐☐ WTE		☐☐ hrs ☐☐ mins
☐ Other *(specify)*	☐☐	☐.☐☐ WTE		☐☐ hrs ☐☐ mins
☐ Other *(specify)*	☐☐	☐.☐☐ WTE		☐☐ hrs ☐☐ mins

Has the child's behaviour disrupted lessons to the point where they had to be removed from class over the last 6 months? *(please cross one box only)*

☐ Yes ☐ No

- How many times? ☐☐☐ times
- On average, how long does it take to stop the disruption in class? ☐☐☐ minutes
